# Regional brain volumes relate to Alzheimer’s disease cerebrospinal fluid biomarkers and neuropsychometry: A cross-sectional, observational study

**DOI:** 10.1371/journal.pone.0254332

**Published:** 2021-07-22

**Authors:** Mark R. Libowitz, Ke Wei, Thao Tran, Karen Chu, Kristina Moncrieffe, Michael G. Harrington, Kevin King

**Affiliations:** 1 Magnetic Resonance Program, Huntington Medical Research Institutes, Pasadena, California, United States of America; 2 Fuller Graduate School of Psychology, Pasadena, California, United States of America; 3 Molecular Neurology Program, Huntington Medical Research Institutes, Pasadena, California, United States of America; 4 Barrow Neurological Institute, Phoenix, Arizona, United States of America; University of California, San Francisco, UNITED STATES

## Abstract

We hypothesized that automated assessment of brain volumes on MRI can predict presence of cerebrospinal fluid abnormal ß-amyloid_42_ and Tau protein levels and thus serve as a useful screening test for possible Alzheimer’s disease. 113 participants ranging from cognitively healthy to Alzheimer’s disease underwent MRI exams to obtain measurements of hippocampus, prefrontal cortex, precuneus, parietal cortex, and occipital lobe volumes. A non-exclusive subset (n = 107) consented to lumbar punctures to obtain cerebrospinal fluid for ß-amyloid_42_ and Tau protein assessment including cognitively health (n = 75), mild cognitively impaired (n = 22), and Alzheimer’s disease (n = 10). After adjustment for false discovery rate, ß-amyloid_42_ was significantly associated with volumes in the hippocampus (p = 0.043), prefrontal cortex (p = 0.010), precuneus (p = 0.024), and the posterior cingulate (p = 0.002). No association between Tau levels and regional brain volume survived multiple test correction. Secondary analysis was performed to determine associations between MRI brain volumes and CSF protein levels to neuropsychological impairment. A non-exclusive subset (n = 96) including cognitively healthy (n = 72), mild cognitively impaired (n = 21), and Alzheimer’s disease (n = 3) participants underwent Stroop Interference and Boston Naming neuropsychological testing. A higher score on the Boston Naming Test was optimally predicted in a selective regression model by greater hippocampus volume (p = 0.002), a higher ratio of ß-amyloid_42_ to Tau protein levels (p < 0.001), greater posterior cingulate volume (p = 0.0193), age (p = 0.0271), and a higher education level (p = 0.002). A better performance on the Stroop Interference Test was optimally predicted by greater hippocampus volume (p = 0.0003) and a higher education level (p < 0.001). Lastly, impaired cognitive status (mild cognitive impairment and Alzheimer’s Disease) was optimally predicted in a selective regression model by a worse performance on the Stroop Interference Test (p < 0.001), a worse performance on the Boston Naming Test (p < 0.001), along with lower prefrontal cortex volume (p = 0.002) and lower hippocampus volume (p = 0.007).

## Introduction

Diagnosis of Alzheimer’s disease (AD) is increasingly focused on discovering biomarkers for early detection [1]. Early detection of AD is crucial as future treatments will likely focus on preventing AD or slowing its progression rather than reversing AD’s neuronal damage [2]. Individuals receiving early diagnosis of pre-clinical AD changes may benefit from initiating health measures to preserve existing cognitive function [1]. Low cerebrospinal fluid (CSF) ß-amyloid_42_ (Aβ_42_) and elevated CSF Tau proteins are two biomarkers that have been established in the 2011 diagnostic guidelines of AD [3]. Previous research identified these two biomarkers as a diagnostic marker of AD in CSF based on Aβ_42_/Tau ratio [4–6] with a lower ratio present in AD pathology as compared to normal pathology. Obtaining CSF for analysis is invasive, however, and positron emission tomography for amyloid or tau involve radiation exposure and are cost prohibitive for screening. MRI examinations of the brain are increasingly common for the workup of memory loss but are not routinely used to screen for risk of dementia. Significant additional value may be provided from these exams if they were used to help suggest presence of AD-related pathology. We hypothesized that reduced CSF Aß_42_ and increased CSF Tau protein levels would show significant association with lower regional brain volumetric assessment.

In addition to CSF biomarkers, determining the relationship between regional brain volume and neuropsychological testing can help define the brain biological changes that underlie AD symptomatology. As a secondary measure, outcomes from two neuropsychological examinations were used in the present study, the Boston Naming Test [7] and the Stroop Interference Test [8]. Boston Naming is a visual confrontation naming test [9, 10] that is used as a test for semantic memory [11]. The Stroop Interference test is commonly used as a measure of executive function [12].

Five brain regions were chosen to test correlation with CSF and cognitive correlates of brain atrophy in AD [9, 13]. These regions include the prefrontal cortex, hippocampus, precuneus, posterior cingulate and, as a negative control, the occipital lobe. The prefrontal cortex is closely linked with cognitive executive functions [14–18] and we specifically predicted it would correlate most closely with performance on the Stroop Interference Test. The hippocampus is involved in registration and retrieval of semantically and lexically associated words [19–22] and was expected to have the closest correlation with the Boston Naming Test. The precuneus was included due to its early degeneration with aging and in early stage AD patients [23] and its association with poor performance on the Montreal Cognitive Assessment in older adults [24]. The posterior cingulate also demonstrates early involvement in preclinical stages of AD [25, 26] with continued neurodegeneration as AD progresses [27]. Finally, the occipital lobe was chosen as a control as it is not heavily involved in early AD pathology. We hypothesized that decreased regional brain volumes and a lower CSF Aß_42_/Tau ratio would suggest presence of deficits on these two sensitive neuropsychological tests of cognitive dysfunction.

Our study therefore aimed to identify the associations between CSF Aß_42_ and Tau protein levels with regional brain volumes and then to evaluate the optimal use of each of these markers in predicting impairments on neuropsychological examination among participants in a brain aging study ranging from cognitively healthy (CH) to AD.

## Materials and methods

### Cohort and overall study design

**Fig 1:**
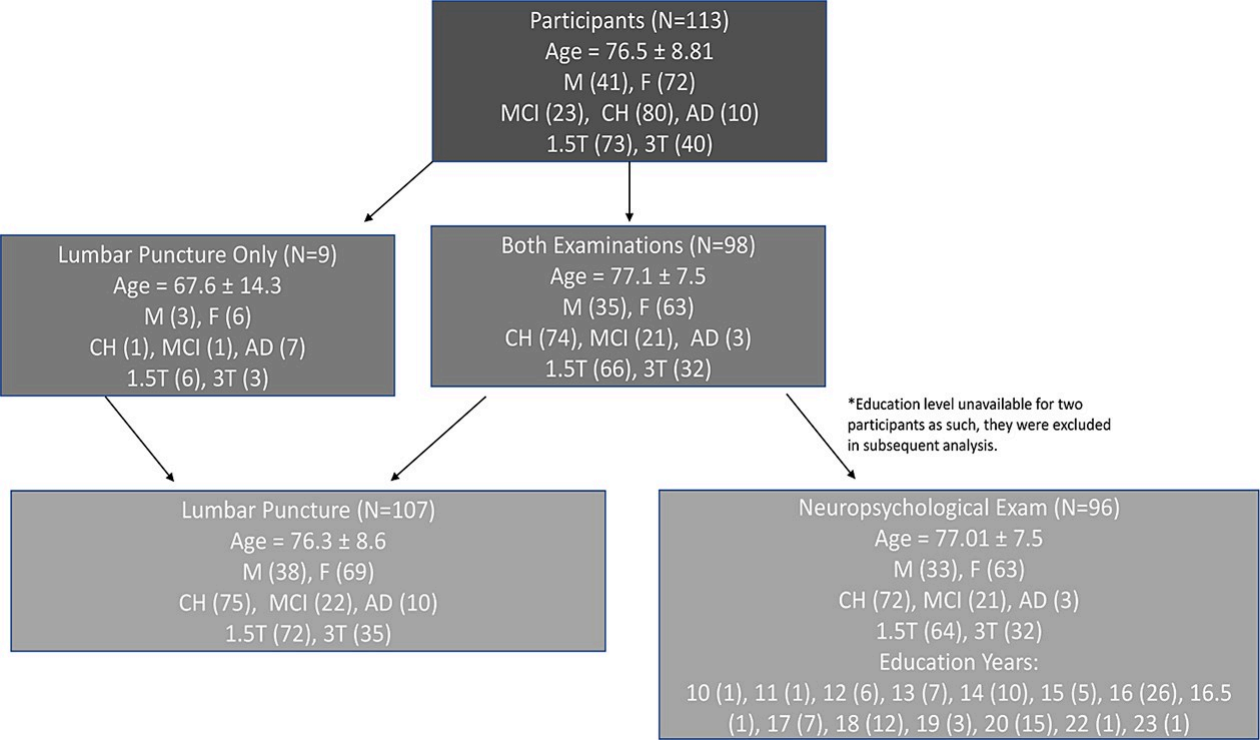
Cohort study design of non-exclusive subsets undergoing lumbar puncture and neuropsychological testing. Flow chart representation of participants included in this study resulting in two non-exclusive subsets. M = Male, F = Female, CH = Cognitively Healthy, MCI = Mild Cognitive Impairment, AD = Alzheimer’s disease, 1.5T = GE Signa HDx 1.5T scanner, 3T = GE Signa HDx 3T scanner.

We evaluated 113 participants (41 males and 72 females, mean age of 76.5 ± 8.8 years) who gave written informed consent in this IRB approved study at Huntington Medical Research Institutes and underwent brain MRI from 2011–2018. Individual participant data is available in the Alzheimer’s Disease Neuroimaging Initiative public database (http://adni.loni.usc.edu/). Most (n = 98) participants underwent both neuropsychological examination and lumbar punctures to obtain CSF for Aβ_42_ and Tau protein assessment. Nine participants underwent lumbar puncture but could not complete cognitive testing (primarily due to presence of dementia) and 2 underwent cognitive testing but did not provide education level, as shown in Fig 1, resulting in 107 with lumbar puncture and 96 with neuropsychological testing. Participant demographics are also listed in Fig 1. Classification of cognitive status was determined in clinical conference with a minimum of three faculty clinicians who reviewed each participant’s results on a battery of neurocognitive exams, MRI and Lumbar Puncture as previously described [4].

### MR image acquisition and analysis

All participants underwent successful MRI examination in either a GE signa HDx 1.5 Tesla or 3 Tesla scanners. 3D fast spoiled gradient echo (FSPGR) were obtained with echo time 2.41ms, repetition time 6.75ms, inversion time 600ms, slice thickness 1.2mm; matrix 256 x 256, FOV 24cm, flip angle 8 degrees. Images were analyzed with FreeSurfer V6 [available for download at http://surfer.nmr.mgh.harvard.edu/] to obtain brain volumes. Prefrontal cortex regions combined the volumes of the frontal pole, superior frontal, caudal middle frontal, rostral middle frontal, lateral orbito-frontal, medial orbito-frontal, pars opercularis, pars orbitalis and pars triangularis regions. Occipital regions combined the volumes of the cuneus, fusiform, lateral occipital, lingual, and pericalcarine regions.

### Cerebrospinal fluid analysis, cognitive status, and neuropsychological examination

CSF Aß_42_ and Tau protein levels were determined via lumbar puncture in 107 participants [4]. Cognitive status was classified at consensus clinical conferencing, including medical history and examination, and an extensive AD neurocognitive battery that tested 6 cognitive domains [4]. Participants were administered the Stroop Interference and Boston Naming Test by supervised and trained graduate neuropsychology students [4].

### Statistics

Analysis was performed using JMP Pro, version 15.0.0 (SAS institute, Inc., Cary, NC). Statistical significance was set at *P* = .05 and two-tailed for all tests. For primary analysis, the p values were adjusted for multiple comparison testing using the Benjamini and Hochberg false discovery rate [28]. All variables were evaluated for normality, and CSF Aβ_42_ and Tau levels were log transformed for statistical analysis to obtain a more normal distribution. Age, sex, intracranial volume and scanner type (GE Signa HDx 1.5T and 3T scanners) were considered as adjustment factors for regional volumetric analysis using general linear models. Residual values for regional brain volumes not explained by age, sex, scanner type and intracranial volume were then derived. The derived equations are listed at the beginning of the results section.

First, we assessed the logarithmic CSF Aβ_42_ and Tau protein levels as independent variables to predict residual values for the dependent variables of the hippocampus, occipital, prefrontal, posterior cingulate, and precuneus volumes. Each analysis underwent false discovery rate adjustment of p-values. We expected both decreased Aβ_42_ and increased Tau CSF protein levels to show association with lower regional volumes for each region besides the occipital lobe.

In secondary analysis, we utilized a stepwise best fit model optimizing stepwise Bayesian Information Criterion (BIC) [29] that included as potential predictors each regional brain volume, log Aβ_42_, log Tau, Aβ_42_/Tau ratio, education level, sex and age with scores on the Stroop Interference and Boston Naming test as the dependent outcome variables. We predicted Boston Naming scores would be higher as a function of greater hippocampus volume and that better Stroop Interference scores would correlate with larger prefrontal volume. Lastly, we utilized a stepwise BIC [29] to determine the best predictor of normal, MCI, and AD consensus cognitive status coded as ordinal categorical variables.

## Results

Regional brain volumes were first adjusted to remove the influence of sex (male = 0, female = 1), scanner type (3T = 0, 1.5T = 1) and intracranial volume using the following equations:

ExpectedHippocampalVolume=5908+43.65×sex+−379.9×scanner+−32.21×age+0.002×intracranialvolume

ExpectedPrefrontalCortexVolume=65400+−798.8×sex+−19240×scanner+−47.27×age+0.040×intracranialvolume

ExpectedPrecuneusVolume=10140+−682.2×sex+−1582×scanner+11.22×age+0.004×intracranialvolume

ExpectedPosteriorCingulateVolume=4783+−185.5×sex+−322.1×scanner+−8.390×age+0.001×intracranialvolume

ExpectedOccipitalVolume=35760+181.9×sex+−7428×scanner+−17.23×age+0.019×intracranialvolume

Lower CSF Aβ_42_ levels were significantly associated with hippocampus (p = 0.017), prefrontal cortex (p = 0.002), precuneus (p = 0.007), posterior cingulate (p = 0.002), and occipital lobe (p = 0.035) volumes. These significant associations withstood false discovery rate adjustment for the hippocampus (p = 0.043), prefrontal cortex (p = 0.010), precuneus (p = 0.024), and posterior cingulate (p = 0.010), but not for the occipital lobe (p = 0.070). Increased CSF Tau protein levels were not significantly associated with any regional brain volume (Table 1).

**Table 1:** Log CSF Aβ_42_ and Tau predicting regional brain volumes adjusted for age, sex, scanner type and intracranial volume.

	Hippocampus		Prefrontal Cortex		Precuneus		Posterior Cingulate		Occipital	
	Parameter Estimate (μL) ± STD	FDR Adj P value	Parameter Estimate (μL) ± STD	FDR Adj P value	Parameter Estimate (μL) ± Standard Deviation	FDR Adj P value	Parameter Estimate (μL) ± Standard Deviation	FDR Adj P value	Parameter Estimate (μL) ± Standard Deviation	FDR Adj P value
CSF Aβ_42_^a^	394±163	0.043	6262±1930	0.01	968±354	0.024	542±171	0.01	na	0.07
CSF Tau^a^	na	0.6	na	0.8	na	0.8	na	0.6	na	0.8

CSF = cerebrospinal fluid, FDR Adj P value = false discovery rate adjusted p value, STD = standard deviation, Aβ_42_ = amyloid-beta_42_ protein, Tau = tau protein.

^a^ logarithmic Aβ_42_ and Tau CSF protein levels.

^b^deviation from expected regional brain volume, 0 μL—adjusted for age, sex, scanner type, and intracranial volume.

^c^*p* values were calculated using analysis of variance model.

For those administered the Boston Naming and Stroop Interference test (N = 96) the best fit model identified higher education (p < 0.001), female sex (p = 0.002), and higher hippocampus volume (p < 0.001) as significant predictors of Stroop Interference scores (p < 0.001; adjusted r^2^ = 0.264) and identified higher education (p = 0.0198), higher hippocampus volume (p = 0.002), higher posterior cingulate volume (p = 0.019), lower Aβ_42_/Tau ratio (p < 0.001), and age (p = 0.0271) as significant predictors of the Boston Naming Test (p < 0.001; adjusted r^2^ = 0.218). Lastly, in a best predictive model minimizing BIC, Stroop Interference Test score (p < 0.001), Boston Naming Test score (p < 0.001), prefrontal cortex volume (p = 0.002), and hippocampus volume (p = 0.007) were the best predictors for presence of MCI or AD (p < 0.001; r^2^ = 0.37). Notably, the Stroop Interference Test was the most significant factor in this model with a p value of 6.5 x 10^−10^.

## Discussion

In order to evaluate a group of participants in a brain aging study at our institution we performed lumbar punctures, MR imaging, and neuropsychological testing. To begin we set out to determine if CSF Aß_42_ or Tau levels were associated with hippocampus, prefrontal cortex, precuneus, posterior cingulate, or occipital lobe volumes. We had predicted that both a decrease in CSF Aß_4_ and an increase in CSF Tau would be associated with all regional brain volumes with exception of the occipital lobe. We found that a decrease in CSF Aß_42_ and not an increase in CSF Tau levels to be significantly associated (surviving a false discovery rate adjustment) with the hippocampus, prefrontal cortex, precuneus, and posterior cingulate. As previously discussed, the ratio of CSF Aß_42_ to Tau protein levels has been utilized as biomarker of AD (citations). The results from our cohort provide evidence that decreased CSF Aß_42_ is more predictive of the regional brain volumes of interest than increased CSF Tau levels. Further, our cohort consisted of participants with a range of cognitive statuses from CH to AD. Therefore, our results suggest that decreased CSF Aß_42_ levels serve as a stronger predictor than increased CSF Tau levels earlier in the process of brain ageing. Finally, CSF Tau levels have been shown to have greater increases as dementia progresses [3], it is likely that this association would survive false discovery rate adjustment as our cohort ages.

Through exploratory analysis we also found that in our cohort several factors were significant predictors of the Stroop Interference and Boston Naming test (Table 2). Smaller brain volumes identified those likely to have deficits on cognitive testing. In order of significance: higher ratio of Aß_42_ to Tau, smaller hippocampus volume, smaller posterior cingulate volume, and education level were all significant predictors of the Boston Naming Test, which assesses semantic memory [11]. Worse performance on the Stroop Interference test was best predicted by education level, low hippocampus volume, and the male sex. Unexpectedly prefrontal cortex volume was not predictive of Stroop Interference score, although it is typically associated with executive functions [12] thought to reside primarily in the frontal lobe.

**Table 2:** Stepwise best fit model reveals best predictors of Stroop interference and Boston naming test raw scores taking into account regional brain volume, CSF protein levels, education level, age, and sex.

	Stroop Interference Test		Boston Naming Test	
Significant Predictor	P-Value^a^	Parameter Estimate and Standard Deviation	P-Value^a^	Parameter Estimate and Standard Deviation
**Hippocampus**	.0003	-0.022 ± 0.006	.0016	0.0013 ± 0.0004
**Education Level**	< .0001	-7.87 ± 1.61	.0198	0.2728 ± 0.1150
**Sex (female)**	.002	-13.99 ± 4.41	na	na
**Posterior Cingulate**	na	na	.0193	-0.0009 ± 0.0004
**AB/Tau Ratio**	na	na	.0008	0.2728 ± 0.1443
**Age**	na	na	.0271	-0.08689 ± 0.03869

^a^*p-value*, parameter estimate, and standard deviation utilizing Bayesian Information Criterion.

Our results show a significant role for the hippocampus in cognitive tests involving both the Stroop Interference and Boston Naming tests. This is concordant with an fMRI study that showed a broad neural network including working memory is required during the Stroop Interference Test [30]. Working memory has also been shown to involve a broad neural network including the dorsolateral prefrontal cortex as well as the hippocampus [30–35]. Future studies should examine whether this network is affected by AD pathology to further establish a link between hippocampus volume and executive function during Stroop Interference testing as demonstrated in this study.

Finally, we found that the lower Stroop Interreference Test score optimally predicted MCI and AD in our cohort which was further aided by lower Boston Naming Test score, prefrontal volume, and hippocampus volume using a selective regression model. Executive function and working memory are known to decline in the progression of MCI and AD (26550575). Considering both the Stroop Interference Test [12] as well as the prefrontal cortex are associated with cognitive executive function [14–18], working memory has shown involvement of a neural network that includes the prefrontal cortex and hippocampus, and the hippocampus was strongly associated with the Boston Naming Test in our cohort it stands to reason that these four factors served as the most optimal predictors of cognitive status. Still considering the evidence of CSF levels of Aß_42_ and Tau protein level’s association with AD we were surprised to find they did not help further improve prediction of cognitive status. Again, our cohort consists of participants with a range of cognitive statuses, thus it may be that these biomarkers would further improve predictions of cognitive status as this cohort ages.

There were several limitations to our study. This is an observational cross-sectional study that does not imply causation. Abnormal amyloid and tau are not specific for AD neuropathology and may occur with other neurodegenerative conditions. We used information from two MRI scanners of different strengths, 1.5 and 3 Tesla which results in a systematic bias in the brain volumes obtained. Differences between scanners is a common barrier to implementation of quantitative standards for brain volumetry. By accounting for the differences between 1.5 and 3 Tesla scans we found there to be a systematic shift that we could isolate and adjust and still identify significant differences with other clinical markers. Crucially, this allowed us to include data from both scanners in each subset. This work helps to show that in spite of technical differences, it is possible to identify meaningful associations in evaluating cohorts assessed on different scanners. This is important as in a clinical setting it is common that a different scanner will be used for a patient or participant and our findings suggest that significant differences in brain volumes may still be identified after appropriate adjustment.

## Conclusion

In this brain aging study, we were able to identify lower MRI regional volumes related to abnormal CSF levels of Aß_42_ but not to Tau; this disparity may be due to amyloid becoming abnormal earlier in the disease course and the lack of individuals with more advanced levels of dementia in this study. MRI regional volumes also showed added utility in predicting cognitive performance: hippocampus and posterior cingulate volume helped improve prediction of Boston naming test score alongside Aß_42_/Tau Ratio; hippocampal volume predicted score on Stroop without any added value for CSF Aß_42_and Tau levels. Interestingly, performance on Stroop interference testing and prefrontal volumes were the best predictors of MCI and AD status in our cohort, with no added predictive value from CSF Aß_42_and Tau levels. This work suggests the importance of future work considering the timing and interdependence of changes in brain volumes and CSF levels of Aß_42_ and Tau in neurodegeneration and impairment of neural networks in understanding deficits in neuropsychological testing.
